# Frequency of early childhood trauma in psychiatric patients: an investigation with the Early Trauma Inventory–Self Report

**DOI:** 10.1192/j.eurpsy.2024.399

**Published:** 2024-08-27

**Authors:** N. M. Szeifert, B. Sebők, B. Szabó, M. Miklósi, Á. Schmelowszky

**Affiliations:** ^1^Doctoral School of Psychology; ^2^Clinical Psychology and Addictology, ELTE Eötvös Lóránd University; ^3^Psychotherapy, National Institute of Sports Medicine; ^4^School of PhD Studies Workgroup for Science Management, Semmelweis University; ^5^Developmental and Clinical Child Psychology, ELTE Eötvös Lóránd University; ^6^Department of Clinical Psychology, Semmelweis University Faculty of Medicine; ^7^Centre of Mental Health, Heim Pál National Pediatric Institute, Budapest, Hungary

## Abstract

**Introduction:**

Childhood trauma is an important public health problem but there are limitations in our ability to measure childhood trauma. Early Trauma Inventory is a self-report instrument for the assessment of childhood trauma that is valid but simple to administer.

**Objectives:**

We aimed to assess the frequency of childhood trauma in patients of a large sample of the Crisis Intervention and Psychiatric Ward in Budapest, Hungary.

**Methods:**

Data from 279 patients referred to Péterfy Alexander Hospital, Crisis Intervention and Psychiatric Ward, Budapest, Hungary, were analyzed. Most participants were female (*n* = 202, 72.4%) between the ages of 17 and 86 (*M* = 38.37 yrs). Half of the participants were diagnosed with major depressive disorder (*n* = 138, 49.5%) or anxiety disorder (*n* = 149, 53.4%), while 47 of the participants suffered from bipolar disorder (16.8%). One hundred thirty-eight participants had at least one suicide attempt in their life (49.5%). Childhood traumas were assessed by the Early Trauma Inventory– Self Report (ETI-SR), an instrument for the assessment of physical, emotional, and sexual abuse, as well as general traumas, which measures frequency, onset, emotional impact, and other variables. We assessed the most frequent traumas in the physical, emotional, and sexual abuse, as well as general trauma domains.

**Results:**

Family mental illness (*n* = 136, 58.1%), witnessing violence (*n* = 129, 54.7%), divorce/separation of parents (*n* = 114, 48.3%), and observing death/serious injury of others (*n* = 112, 47.5%), were the most frequently experienced general traumas. Out of physical traumas, most of the participants experienced being slapped in the face (*n* = 169, 73.2%), being spanked with a hand (*n* = 152, 65.5%), being hit or spanked with an object (*n* = 93, 40.3%), and being pushed or shoved (*n* = 81, 33.4%). Among emotional traumas, being often put down or ridiculed (*n* = 170, 74.2%), the needs being failed to be understood by parents (*n* = 164, 72.7%), often shouted at or yelled at (*n* = 154, 67.5%), and being often ignored or made feel like they do not count (*n* = 109, 46.2%) were the most frequent. From the sexual abuse domain, being exposed to flashing (*n* = 72, 32.9%), being touched in intimate parts in an uncomfortable way (*n* = 63, 29.2%), being exposed to inappropriate comments about sex (*n* = 61, 28.5%), and being rubbed by someone’s genitals (*n* = 44, 20.3%) were the most common. Further results will be presented at the conference.

**Conclusions:**

It is already recognized and our study also confirms that childhood maltreatment, especially sexual abuse can lead to suicidal behaviour. The precise role of particular types of childhood maltreatment and the mediators of the relationship between childhood maltreatment and suicide is yet to be investigated in more details.

**Disclosure of Interest:**

None Declared

